# The International Mouse Phenotyping Consortium (IMPC): a functional catalogue of the mammalian genome that informs conservation

**DOI:** 10.1007/s10592-018-1072-9

**Published:** 2018-05-19

**Authors:** Violeta Muñoz-Fuentes, Pilar Cacheiro, Terrence F. Meehan, Juan Antonio Aguilar-Pimentel, Steve D. M. Brown, Ann M. Flenniken, Paul Flicek, Antonella Galli, Hamed Haseli Mashhadi, Martin Hrabě de Angelis, Jong Kyoung Kim, K. C. Kent Lloyd, Colin McKerlie, Hugh Morgan, Stephen A. Murray, Lauryl M. J. Nutter, Patrick T. Reilly, John R. Seavitt, Je Kyung Seong, Michelle Simon, Hannah Wardle-Jones, Ann-Marie Mallon, Damian Smedley, Helen E. Parkinson, the IMPC consortium

**Affiliations:** 1 European Molecular Biology Laboratory, European Bioinformatics Institute (EMBL-EBI), Wellcome Genome Campus, Hinxton, Cambridge, CB10 1SD UK; 2 Clinical Pharmacology, William Harvey Research Institute, School of Medicine and Dentistry, Queen Mary University of London, Charterhouse Square, London, EC1M 6BQ UK; 3 German Mouse Clinic, Institute of Experimental Genetics, Helmholtz Zentrum München, German Research Center for Environmental Health, Ingolstädter Landstrasse 1, 85764 Neuherberg, Germany; 4 Medical Research Council Harwell Institute (Mammalian Genetics Unit and Mary Lyon Centre), Harwell, Oxfordshire OX11 0RD UK; 5 The Centre for Phenogenomics, Toronto, ON M5T 3H7 Canada; 6 Mount Sinai Hospital, Toronto, ON M5G 1X5 Canada; 7 Wellcome Trust Sanger Institute, Cambridge, CB10 1SA UK; 8 German Center for Diabetes Research (DZD), Ingolstädter Landstr. 1, 85764 Neuherberg, Germany; 9 School of Life Science Weihenstephan, Technische Universität München, Alte Akademie 8, 85354 Freising, Germany; 10 Department of New Biology, DGIST, Daegu, 42988 Republic of Korea; 11 Mouse Biology Program, University of California, Davis, CA 95618 USA; 12 The Hospital for Sick Children, Toronto, ON M5G 1X84 Canada; 13 The Jackson Laboratory, Bar Harbor, ME 04609 USA; 14 PHENOMIN-iCS, 1 Rue Laurent Fries, 67404 Illkirch Cedex, Alsace France; 15 Department of Molecular and Human Genetics, Baylor College of Medicine, Houston, TX 77030 USA; 16 Laboratory of Developmental Biology and Genomics, College of Veterinary Medicine, Interdisciplinary Program for Bioinformatics and Program for Cancer Biology, Seoul National University, Seoul, Republic of Korea

**Keywords:** Cheetah, Endangered species, Loss-of-function, Non-model species, Panda, Polar bear, Phenotype, Wolf, Essential genes, IMPC, Knockout, Mouse

## Abstract

The International Mouse Phenotyping Consortium (IMPC) is building a catalogue of mammalian gene function by producing and phenotyping a knockout mouse line for every protein-coding gene. To date, the IMPC has generated and characterised 5186 mutant lines. One-third of the lines have been found to be non-viable and over 300 new mouse models of human disease have been identified thus far. While current bioinformatics efforts are focused on translating results to better understand human disease processes, IMPC data also aids understanding genetic function and processes in other species. Here we show, using gorilla genomic data, how genes essential to development in mice can be used to help assess the potentially deleterious impact of gene variants in other species. This type of analyses could be used to select optimal breeders in endangered species to maintain or increase fitness and avoid variants associated to impaired-health phenotypes or loss-of-function mutations in genes of critical importance. We also show, using selected examples from various mammal species, how IMPC data can aid in the identification of candidate genes for studying a condition of interest, deliver information about the mechanisms involved, or support predictions for the function of genes that may play a role in adaptation. With genotyping costs decreasing and the continued improvements of bioinformatics tools, the analyses we demonstrate can be routinely applied.

**Electronic supplementary material:**

The online version of this article (10.1007/s10592-018-1072-9) contains supplementary material, which is available to authorized users.

## The IMPC: a functional catalogue of the mammalian genome

The goal of the International Mouse Phenotyping Consortium (IMPC, http://www.mousephenotype.org) is to generate a functional catalogue of the mammalian genome by producing a knockout mouse line for every protein-coding gene. This is achieved by characterising the phenotypes of mutants and controls, which increases our understanding of development and gene function, and identifies models for disease. Knockout mouse lines are produced on a uniform genetic background using either gene targeted embryonic stem cells (Skarnes et al. 2011) or, increasingly, nuclease-mediated genome editing with CRISPR/Cas9-based methods (Singh et al. 2015; Mianne et al. 2017). A uniform genetic background across controls and mutant lines is necessary to allow for reproducible and comparable results. Some phenotypes will be strongly influenced by the genetic background and, therefore, this is an important consideration to take into account, particularly when translating mouse findings (inbred) to other species (outbred, or mostly outbred; see Discussion). Mice are characterised across a dozen research centres in a standardized phenotyping pipeline (IMPReSS, the International Mouse Phenotyping Resource of Standardised Screens) that includes strict data quality standards and requires the minimum number of animals necessary to achieve statistical significance for each test (Hrabe de Angelis et al. 2015). The IMPC data is integrated and reviewed, and statistically significant outlier phenotypes for individual lines are annotated using PhenStat (Kurbatova et al. 2015) and the Mammalian Phenotype Ontology (MPO) (Beck et al. 2009; Smith and Eppig 2015), which is actively developed to capture phenotypes of mutant mouse lines by Mouse Genome Informatics (MGI) based at Jackson Laboratory. All raw data, results of statistical pipelines and curated phenotype data are made publicly available through the IMPC website. The data are further integrated with other resources, including OMIM, MGI and Ensembl. The IMPC database is searchable by gene name, phenotype and disease, allows batch queries and the download of all data, dedicated reports, graphs and images.

To date, 5186 mutant lines have been phenotyped (data release 7.0), with an average of 163 parameters measured on any given mouse, represented by over 128,000 knockouts and 35,000 wildtype or control mice. In addition, embryonic lethal mouse lines are analysed in a specialized embryonic development pipeline that utilizes high-resolution 3D imaging to understand structural changes (Dickinson et al. 2016). These data allow the IMPC to identify the physiological systems that are disrupted when a gene is disabled and make new gene-phenotype associations. Evolutionary conservation of fundamental processes governing development and support of metazoan life allows functional knowledge gained in one species to be translated to others (Kirschner and Gerhart 2006; Liao et al. 2006; Saenko et al. 2008; Bellen et al. 2010; Greek and Rice 2012). The IMPC uses its new gene-phenotype associations to identify models for human disease based on phenotypic similarity scores using PhenoDigm (Smedley et al. 2013), which establishes a link between IMPC mouse phenotypes mapped to the Mammalian Phenotype Ontology and the clinical descriptions of human diseases, as featured in OMIM and Orphanet, mapped to terms of the Human Phenotype Ontology (Kohler et al. 2017). Based on data from 3,328 genes, 360 new disease models have so far been identified by the IMPC, allowing researchers to investigate molecular mechanisms underpinning human genetic diseases, and explore new routes of therapeutic intervention (Meehan et al. 2017). While the IMPC has focused on translating knowledge from mouse to human, the translation to other species, including wild and endangered, is relevant as well.

## Wild species may benefit from functional knowledge accumulated in the laboratory mouse

Endangered species typically suffer dramatic declines before remedial measures are put into place. During a species decline, genetic erosion results in the loss of genetic variation that limits a species’ ability to adapt to changes in the environment and increases the chances for the accumulation of deleterious mutations that affect reproduction and fitness. Fertility-related disorders have been documented in the African cheetah, *Acinonyx jubatus* (Wildt et al. 1983; Crosier et al. 2007), the Florida panther, *Puma concolor coryi* (Roelke et al. 1993; Johnson et al. 2010) and the Iberian lynx, *Lynx pardinus* (Ruiz-Lopez et al. 2012). Similarly, bone and dental anomalies have been observed in inbred wolf (*Canis lupus*) populations in Isle Royale in North America and Scandinavia (Raikkonen et al. 2009, 2013). In an attempt to reverse these situations and decrease inbreeding, breeding with closely related species has been implemented in the case of the Florida panther and the puma *Puma concolor stanleyana* (Johnson et al. 2010). These genetic rescue approaches need to be carefully considered, as they may cause increased inbreeding as well as loss of species-specific adaptations (Hedrick and Fredrickson 2010), and even forfeiture of legal protected status, e.g., Endangered Species Act (Haig and Allendorf 2006). Clearly, identifying the critical genes associated with disorders as well as species-specific adaptations is important from a conservation perspective to maximise conservation of adaptive potential and, if needed, preserve genetic fitness through selective breeding.

The genomes of many mammals have been sequenced in the last 15 years. We selected a number of mammalian species for which functional adaptations have been explored and illustrate how knockout mouse phenotype information can support or complement predictions for the function of genes that may play a role in adaptation, provide a panel of genes for studying a phenotype of interest, or aid deciphering the mechanisms involved in underlying certain conditions.

## Essential genes in mice and humans: mining wildlife genomes for LoF gene variants to identify basis of reduced fitness—a pilot study

A previous analysis of IMPC’s high-throughput mammalian embryonic phenotype data for 1751 knockout mouse lines resulted in 24, 11 and 65% of the lines being associated to a lethal, subviable and viable phenotype, respectively; this led to the conclusion that, in mice, approximately 35% of the genes are essential for organism viability (Dickinson et al. 2016). We hypothesize that these genes are essential in other mammalian species, and variants causing loss of function in these critical genes might, therefore, be undesirable. To test this hypothesis, we first compared genes identified as essential in IMPC mice with those identified in humans based on cell viability. We then used these essential genes to gain further insight into loss-of-function (LoF) variants (protein-coding genes containing substitutions that introduce a stop codon, frameshift indels, or modifications of essential splice sites) identified in inbred populations of gorillas.

The IMPC viability screen identifies genes essential for organism viability by identifying mouse lines which are lethal (absence of homozygote pups for the knockout allele or homozygote null pups), subviable (the frequency of homozygote null pups is less than 12.5%, or less than 50% of the 25% predicted in a heterozygote × heterozygote crossing) or viable (all others). We conducted an updated analysis on viability data for 4237 genes currently available in IMPC DR7.0, which included the 1751 previously analysed in Dickinson et al. (2016) (see Supplementary Methods). We found that 25, 9, and 66% of the lines resulted in a lethal, subviable and viable phenotype, respectively (Supplementary Datafile S1), nearly identical proportions to those reported in Dickinson et al. (2016) (see above). These results support the conclusion that about one-third of the genes are essential for life, as described in an earlier publication surveying the knockout mouse literature (Adams et al. 2013).

Screens of knockout human cells have identified ~ 2000 genes essential for cell viability in studies of 11 cell lines (Blomen et al. 2015; Hart et al. 2015; Wang et al. 2015). Combining these data sets, 18,862 genes were unequivocally mapped to their HUGO Gene Nomenclature Committee (HGNC) identifiers, of which 17,675 were studied in > 50% of the cell lines (at least 6 cell lines). We defined a set of core essential genes comprising 1568 genes (9%) which were essential for viability in over 50% of the cell lines where the gene was studied. To understand how gene essentiality compares between human cells and mice, we inferred mouse-to-human orthologues and looked at their distribution in the IMPC and human-viability categories. We obtained a dataset containing 4115 IMPC mouse-to-human orthologues (see Supplementary Methods), of which 4026 were included in the human cell studies (Supplementary Datafile S2). We found that 36% of the mouse genes identified as embryonic lethal (i.e., essential) corresponded to genes identified as essential in the human cell lines, while 64% corresponded to genes that are non-essential in cells. In the case of genes identified as embryonic viable in mice (i.e., non-essential), almost all (99.6%) were associated with non-essentiality in the human cell lines (Table 1). These results indicate a strong correspondence between non-essential genes and that about two-thirds as many genes are essential for organismal than for cell viability.

**Table 1:** Overlap of mouse IMPC lethal and viable genes (DR7.0) and human cell essential and non-essential genes

Overlaps		Number of genes
Mouse	Human cell lines^a^	
Lethal	Essential	353 (35.9%)
Lethal	Non-essential	631 (64.1%)
Viable	Essential	9 (0.4%)
Viable	Non-essential	2499 (99.6%)

^a^Essential: genes essential for cell viability in > 50% of the cell lines and studied in > 50% of the cell lines (that is, equivalent to ≥ 6 cell lines)

We then investigated the critical importance of LoF variants in gorillas *Gorilla gorilla*, western Africa, and *G. beringei*, eastern Africa; Xue et al. (2015). Notably, homozygous LoF alleles were found in 241 genes in apparently healthy individuals, and we determined which of these genes are identified as essential in mice or humans. We inferred gorilla-to-mouse orthologues (Supplementary Methods) and obtained a mouse orthologue for 169 out of the 241 gorilla genes, resulting in 192 mouse genes (due to one-to-many conversions, Datafile S3). Western lowland gorillas (*G. g. gorilla*) had 136 homozygous LoF orthologues, eastern lowland gorillas (*G. b. graueri*) had 81, and mountain gorillas (*G. b. beringei*) had 84. Overlap with the viability data obtained by the IMPC (reported above) indicated a distribution of the LoF alleles in the three viability categories similar to that obtained for any protein-coding gene in the IMPC catalogue (Table 2). The percentage of lethal genes in the gorilla populations was lower than in the IMPC viability data (14–24% vs 25%), but the difference was not significant (*P* = 0.731, *P* = 0.659 and *P* = 0.130 for mountain, eastern lowland and western lowland gorillas, respectively, Table S1).

**Table 2:** Mouse orthologues with homozygous LoF alleles as identified by Xue et al. (2015) and their association to a lethal, subviable or viable phenotype based on viability data collected by the IMPC (DR7.0)

	Sample size	Lethal (*n* = 1052)	Subviable (*n* = 383)	Viable (*n* = 2802)
Mountain gorillas (Gbb)	7	5 (24%)	3 (14%)	13 (62%)
Eastern lowland gorillas (Gbg)	9	4 (19%)	3 (14%)	14 (67%
Western lowland gorillas (Ggg)	27	5 (14%)	6 (16%)	26 (70%)
Total (unique)	43	9 (18%	6 (12%)	36 (70%)

Sample size refers to the number of gorillas as indicated in the original publication

**Fig. 1:**
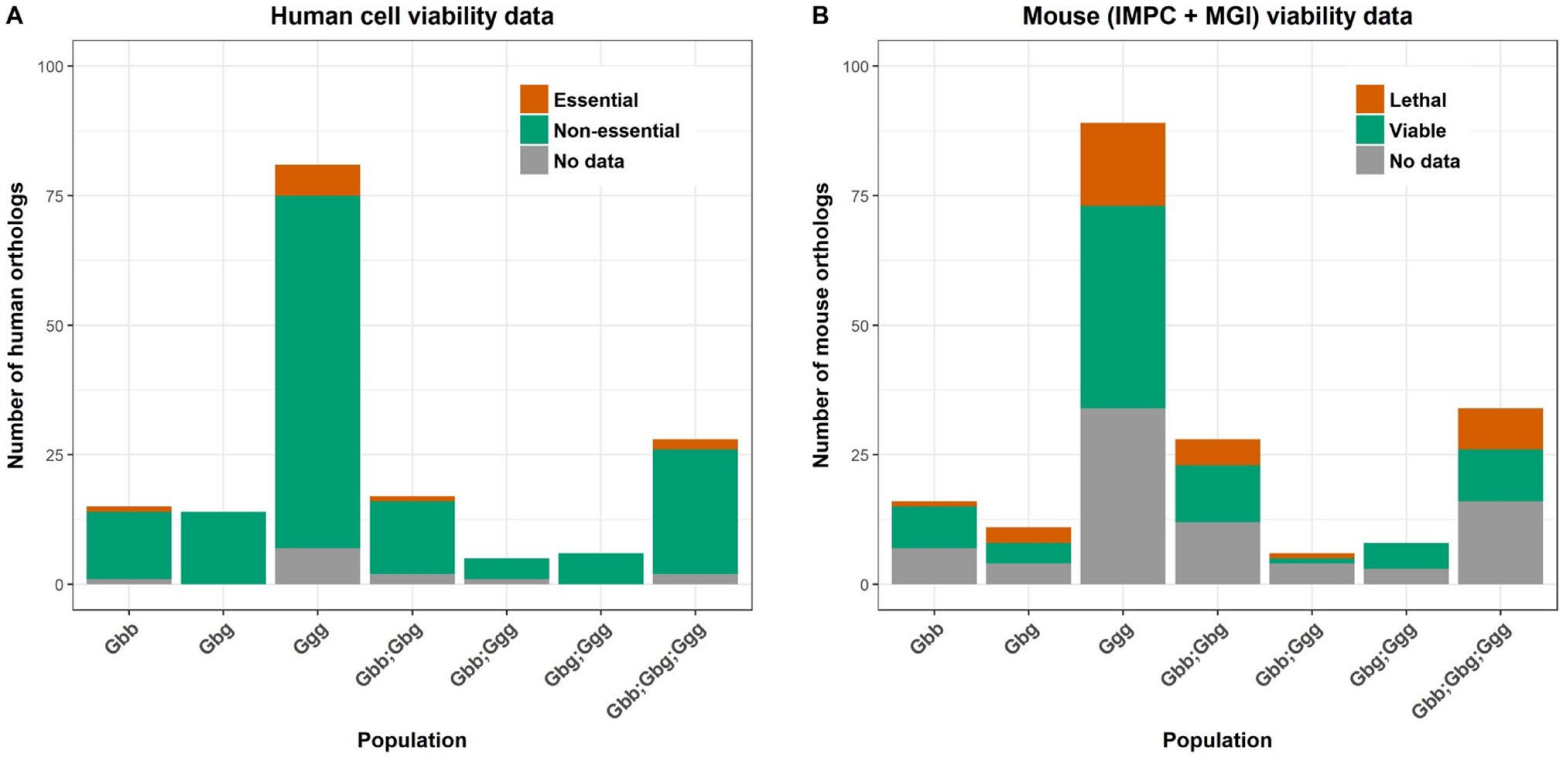
Human (**a**) and mouse orthologues (**b**) of gorilla genes with homozygous LoF alleles and their association to essentiality based on human cell studies (**a**) or IMPC and MGI data (**b**). (Data in Supplementary Table S3). Gorilla populations, from larger to smaller size in the wild: mountain gorillas (Gbb), eastern lowland gorillas (Gbg) and western lowland gorillas (Ggg)

We then proceeded to gain a better understanding of the potential phenotypic impact of the LoF mutations in gorillas. First, we obtained gorilla-to-human orthologues (168 human genes, Datafile S4) and assessed their essentiality using the data from the human cell studies (Fig. 1a). We found that between 5–7% of the genes were essential for cell survival, lower than what would be expected for any gene selected at random (9%), but the difference was not significant (*P* = 0.818, *P* = 0.369 and *P* = 0.736 for mountain, eastern lowland and western lowland gorillas, respectively, Table S2). When bringing in IMPC and MGI phenotypes, the data become increasingly complex and more difficult to interpret. For the 191 mouse orthologues, there was phenotype information for 62% of the genes, and 34% of the genes were associated with an embryonic lethal phenotype (Fig. 1b). It is important to note that any given gene may not be associated with a lethal phenotype in all mouse lines, but be linked to a variety of health-impaired phenotypes that do not cause lethality in additional lines (Datafile S3). For example, homozygotes of *Chd2* investigated in three genetic backgrounds resulted in postnatal lethality in two of them and in viable individuals in the other, but with health-impaired phenotypes associated to growth, the skeleton and the hematopoietic system. These effects can be due to potential differences in genomic modifiers between different strains used to generate the knockouts. Further, most knockouts, including the IMPC ones, are on inbred backgrounds, while wild species will be outbred, or at least more so than laboratory mouse strains. Based on these results, it is therefore possible that gorillas carrying these variants may present clinical complications that may impact their fitness and thus it will be desirable to reduce the prevalence of these alleles, particularly in a recovery population. Alternatively, these truncated variants identified in gorillas may not be affecting the functional exon of the protein or may correspond to genes redundant in function. Although viable lines are more likely to have a paralogue than lethal lines, there are nevertheless some essential genes with paralogues (White et al. 2013; Dickinson et al. 2016) and it is possible that these provide functional compensation for the effect of LoF variants in the inactivated genes. Further research is needed to clarify this situation, including advances to detect pseudogenes e.g. Claes and De Leeneer (2014). Humans carry LoF variants (MacArthur et al. 2012) at about ~ 100 putative variants per individual (The Genomes Project 2012) and the identification of both deleterious and beneficial variants has fuelled significant interest in these regions (Balasubramanian et al. 2017).

## IMPC aids the functional annotation of regions putatively targeted by positive selection to understand the genomic basis of adaptation

**Fig. 2:**
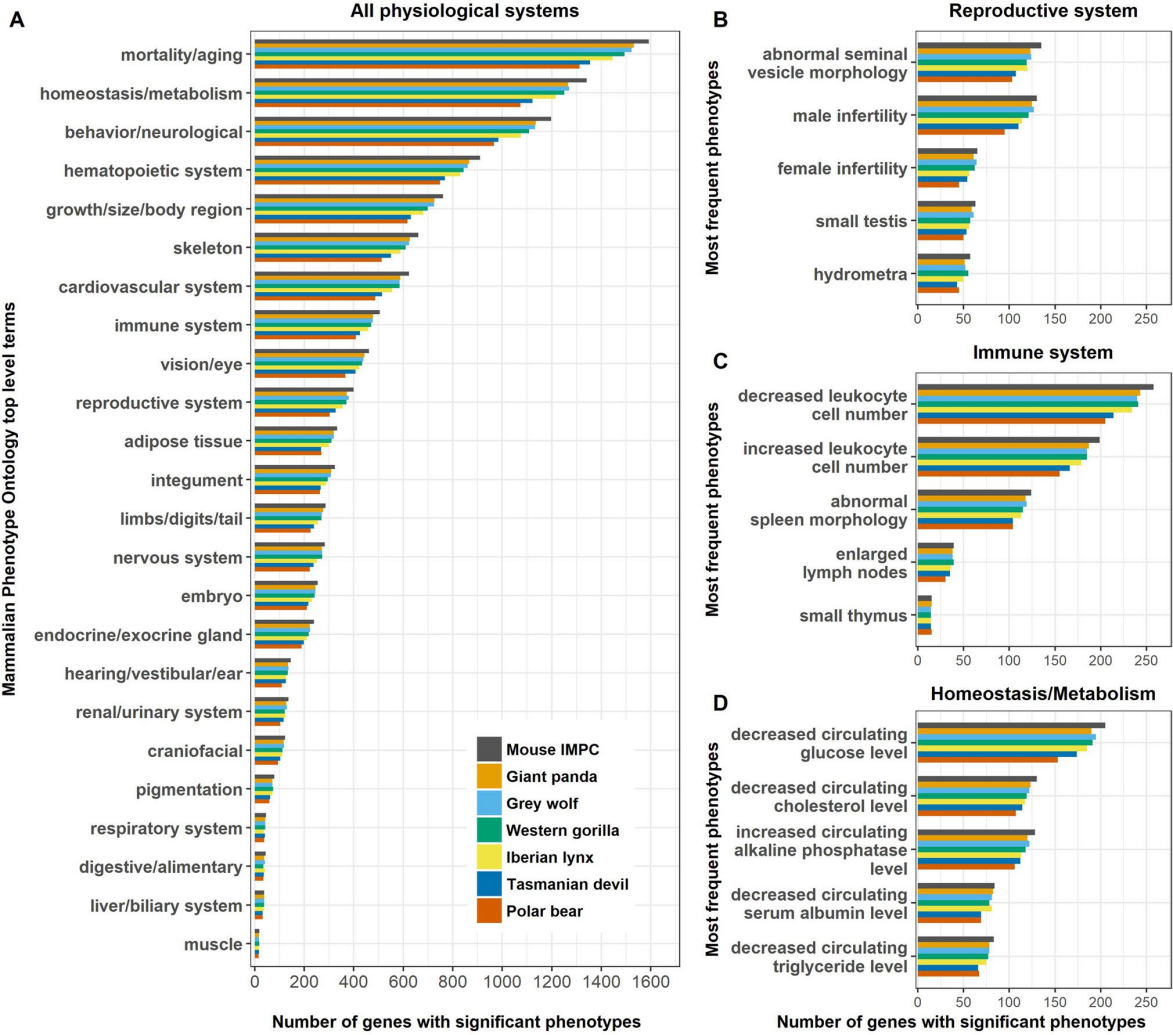
Number of IMPC significant phenotypes for selected mammalian species. A mouse orthologue was found for 71–91% of the genes of each species, of which 24–25% had IMPC phenotype information (DR6.0, Supplementary Table S4). **a** Phenotypes classified according to the top levels of the Mammalian Phenotype Ontology. **b**–**d** Phenotypes can be classified for more granular ontology terms

A number of recent studies in which mammalian gene function and adaptations are evaluated allow us to illustrate additional ways in which the IMPC may constitute a useful resource for mammals other than humans. The African cheetah (*Acinonyx jubatus*), a species with remarkably low levels of genome diversity relative to other mammals, exhibits signs of inbreeding depression in captive and free ranging populations, including low fecundity and malformed spermatozoa (Dobrynin et al. 2015). An initial panel of 964 human genes with gene ontology (GO) terms associated to reproduction, yielded a set of 18 genes with accelerated rate of non-synonymous to synonymous substitution (dN/dS) accumulation in the cheetah lineage and damaging mutations previously associated to reproductive impairment (Dobrynin et al. 2015). We found a mouse orthologue for all genes, 5 with an IMPC significant phenotype (DR6.0, Datafile S5). Two had phenotypes associated with reproduction. One of them, *Rspo1*, was characterized with abnormal morphology in seminal vesicles and testes, small testes, lacZ expression in the *vas deferens* and the epididymis. In addition, this gene is associated with at least one infertility-related disease, progesterone resistance (affecting females). MGI (http://www.informatics.jax.org/, accessed 17 November 2017) had phenotype information for 14 genes, of which 12 were related to the reproductive system. With only about ~ 25% of the protein-coding genes in the mouse genome explored, the IMPC currently contains around 400 mouse genes with phenotypes associated with the reproductive system (Fig. 2b), a potential useful resource to inform future studies on the genetic contributors to low fecundity.

In individuals belonging to the three subspecies of gorillas (*G. g. gorilla, G. b. graueri* and *G. b. beringei*) in the above-mentioned study, polymorphisms were identified in 23 genes corresponding to human disease-causing variants, significantly enriched for blood coagulation phenotypes, with 3 (*TNNT2, KCNE1, PKP2*) associated to cardiomyopathies (Xue et al. 2015). Indeed, cardiovascular disease is an important cause of death for gorillas in captivity (McManamon and Lowenstine 2012). Mouse orthologues were obtained for all except one gene, of which 5 have IMPC phenotype information (*F13A1, HNF4A, KLKB1, NPC1, SHH*), two associated to a cardiovascular phenotype, one to a skeleton phenotype and two to pre-weaning lethality in homozygotes, respectively (Datafile S6). The MGI database included 20 of these genes, of which 11 were associated to cardiovascular phenotypes and 5 to the hematopoietic system. In the IMPC, there are predictions for the association of 585 mouse orthologues of Western gorilla genes to cardiovascular phenotypes, and 844 to hematopoietic system phenotypes (Fig. 2a), potentially constituting an important resource for understanding cardiomyopathies in gorillas.

A study on speciation and adaptation in polar bears (*Ursus maritimus*) identified 20 genes as strong candidates to have been positively selected in polar bears, in what is a prime example of speciation through adaptation to an extreme environment (Liu et al. 2014). The authors reported disease associations in humans and other mammalian model organisms, including mice, suggesting a function for 11 of these genes associated to adipose tissue development and fatty acid metabolism (*APOB*), cardiovascular function (*APOB, ABCC6, ALPK3, ARID5B, CUL7, EHD3, TTN, VCL, XIRP1*) or white fur pigmentation (*LYST, AIM1*), which may be advantageous in the Arctic. Information derived from the IMPC and MGI databases (Datafile S7) supported these predictions and provided evidence for new roles. An exception was *AIM1*(currently *CRYBG1*). IMPC data indicates no association with coat colour or pigmentation. Homozygotes for *AIM1* were not viable, and the heterozygotes presented phenotypes associated with vision and the nervous system, but the fur was normal. In addition, there were phenotypic associations for 3 genes out of the 9 for which no function was reported in the paper. Knockouts for *COL5A3, LAMC3* and *SH3PXD28* presented a variety of phenotypes, including associations with adipose tissue, the cardiovascular and the immune systems and homeostasis, which are functions that the authors indicated might be relevant in adapting to the Arctic.

A recent study on grey wolves (*Canis lupus*) from North America aimed to identify candidate genes under selection and environmentally driven functional variation (Schweizer et al. 2016a, b). In this study, nonsynonymous mutations were significantly correlated with environmental variables in genes associated to lipid metabolism (*APOB, LIPG*), immunity (*DLA-DQA, DLA-DRB1*), olfaction (*OR4S2, OR5B17, OR6B1*), vision and hearing (*PCDH15, USH2A*), and pigmentation (*TYR, TYRP1*), where 4 genes had variants with predicted deleterious impact *LIPG, OR4S2, OR5B17, USH2A* (Schweizer et al. 2016a). Information derived from the IMPC database for 6 of these genes and the MGI database for 23 of them reproduces previous findings and provides evidence for new roles (Datafile S8). *LIPG* is reported to be associated with the metabolic and cardiovascular systems, *USH2A* with the nervous system but also with vision and hearing, and no information is available for genes *OR4S2* and *OR5B17*, potentially related to olfaction.

We identified at least one study focusing on wild species, giant and red pandas *Ailuropoda melanoleuca* and *Ailurus fulgens* (Hu et al. 2017) and two on cattle (Kadri et al. 2014; Biase et al. 2016) that have used the mouse knockout database information to further characterize genes or processes. The panda species are predicted to have independently acquired adaptations in 70 genes to a bamboo-rich diet, including a pseudothumb (limb development genes *DYNC2H1* and *PCNT*) and features related to digestion and nutrient utilization (in particular genes *GIF, CYP4F2, ADH1C* and *CYP3A5*). The IMPC database complements data collected by MGI by providing information for 5 additional genes (Datafile S9). In the two cattle studies, mouse knockout data informed about processes related to infertility (Kadri et al. 2014; Biase et al. 2016).

## Outlook

Here we show how viability data collected by the IMPC are defining a set of essential genes that are likely also relevant in other species, particularly mammals. Identifying deleterious mutations is important for the design of captive breeding strategies (Bosse et al. 2015), and we encourage exploring the potential of the analyses presented here to identify critical functional variants. An assessment of human and mouse genes orthologous to gorilla genes containing homozygous LoF variants indicates that a number of them are strong candidates to compromise fitness and, therefore, further investigation of the phenotypes of gorillas with these variants will be required. The phenotypic effects of LoF or any other variants will manifest under certain genetic conditions (genetic background) or environmental conditions. While a number of phenotypes in the mouse have shown to correlate directly with humans (e.g., Brophy et al. 2017; Santiago-Sim et al. 2017), these findings should, in general, be taken as indicative of directions for further investigations. Currently, mouse outbred stocks that are genetically heterogeneous and diverse, and thus more appropriately mimicking human or wild animal populations, are being used for mapping genes and quantitative trait loci (QTLs) (Winter et al. 2017). Additionally, the prediction of LoF variants is not straightforward and improved methods are under development.

We have shown that the IMPC, by elucidating mammalian gene function, provides experimental evidence to support novel or previously hypothesised relationships between gene function and processes, and aids in characterising hereditary diseases in mammalian species other than human. The IMPC is focusing on characterizing many of the poorly understood genes (the *ignorome*). It is is also making relevant contributions to our understanding of mammalian gene function, in terms of sexual dimorphism (Karp et al. 2017; Rozman et al. 2018), pleiotropy (Brown et al. 2018) and disease (Bowl et al. 2017; Meehan et al. 2017; Perez-Garcia et al. 2018; Rozman et al. 2018). Formidable challenges remain ahead, including understanding, for example, incomplete penetrance, co-regulation of promoters or gene networks and the function of non-coding sequences, especially ultra-conserved non-coding regions that are more highly conserved across species than most protein-coding genes. Recognizing the effects of processes such as epistasis and hitchhiking of variants closely linked to selected genes in the wild species genomes pose a challenge. Another obvious challenge will be to determine gene function of wildlife phenotypes not present in human and mouse (e.g. aquatic phenotypes) and the co-opting of gene function for other biological processes via gene duplication.

The identification of critical functional variants can be of particular importance for endangered species or bottlenecked populations to aid attempts to reduce the incidence of genetically-determined traits that decrease fitness, or limit recovery. However, the benefits of a genetic rescue approach would require that the conditions that led to the accumulation of deleterious alleles are removed from the population. In the case of endangered species with low effective population sizes, the effect of genetic drift will be much greater than that of natural selection. Hence, even with an optimized breeding program in place, the potential gains of selecting critical functional variants in the breeders might be offset by the stochastic effects of genetic drift. When attempting to develop strategies to preserve adaptive variation, a design where breeding can be managed closely might be desired. For example, the establishment of a captive insurance metapopulation for the Tasmanian devil aims at maximising genetic diversity and keeping a healthy stock of individuals that can be used as a source population for re-wilding and genetic rescue (Gooley et al. 2017).

Advances in genotyping and whole-genome sequencing are resulting in an increase in the number of available genomes and transcriptomes, as well as improved methods to analyse these data, infer orthologous relationships and generate cross-species knowledge. In addition, integration of phenotype data is expected to become prominent in evolutionary studies. In order to produce databases that are computationally tractable and that allow for cross-species integrations, as well as to avoid loss of information, adhering to standards and persistent genetic identifiers (e.g., Ensembl, HGNC or MGI identifiers), as well as applying purpose-oriented ontologies, will be critical. In evolutionary biology and phylogenetic systematics, efforts to computationally integrate genetic, phenotypic and anatomical data include the ‘Phenotype And Trait Ontology’ (PATO; Mabee et al. 2007) and the Phenoscape project (Dahdul et al. 2010) but improvements in this area will certainly be needed (McMurry et al. 2017).

Animal models have proved useful to develop assisted reproductive technologies for endangered species, including lessons learned from oocyte and embryo culture in domestic animals and humans, and oncofertility techniques applied to humans (Comizzoli et al. 2010). Recently, cryopreservation of gametes was used to recover past genetic diversity in the black-footed ferret (*Mustela nigripes*; Wildt et al. 2016) and in vitro fertilization of frozen oocytes and spermatozoa is now the only way in which the northern white rhino (*Ceratotherium simum cottoni*) may be rescued (Saragusty et al. 2016). Studies on domestic mammals provide molecular markers that can be transferred for use in non-model species to inform about molecular processes with potentially phenotypic implications (Munoz-Fuentes et al. 2015). Moreover, understanding consequences of gene variants in other species may be of importance for human health and disease; for example, polar bears have evolved adaptations to deal with extremely fat-rich diets (see above), which are a major concern in human health. Currently, methods based on genomic data are being put forward to improve breeding strategies of wild species to attempt to minimize the impact of undesirable genetic variants while maintaining acceptable levels of genetic diversity (Bosse et al. 2015; Irizarry et al. 2016) and rapid advances in CRISPR/Cas9 technology in animal models to reduce the risk of off-target mutagenesis opens up opportunities to eliminate deleterious mutations in zygotes. In the case of wild species, such methods would allow the persistence of fitness-linked alleles and the avoidance of deleterious mutations without the risks associated with inbreeding or breeding between two similar species. The combined accumulation of gene function annotation by the IMPC and their advances in the use of CRISPR/Cas9 technology will be able to assist in future conservation efforts.

## Electronic supplementary material

Below is the link to the electronic supplementary material.

Supplementary material 1 (PDF 166 KB)

Supplementary material 2 (XLSX 829 KB)
