# Supplementary material for: The International Mouse Phenotyping Consortium (IMPC): a functional catalogue of the mammalian genome that informs conservation
Source: Conserv Genet. 2018 May 19;19(4):995–1005. doi: 10.1007/s10592-018-1072-9 (PMC6061128; doi:10.1007/s10592-018-1072-9)
Supplement: Supplementary file 1 — Supplementary material 1 (PDF 166 KB) [file 10592_2018_1072_MOESM1_ESM.pdf]

Supplementary Information

The International Mouse Phenotyping Consortium (IMPC): a functional catalogue of the mammalian genome that informs conservation

Violeta Muñoz-Fuentes,[1ξ*] Pilar Cacheiro,[2ξ] Terrence F Meehan,[1] Antonio Aguilar-Pimentel,[3] Steve D. M. Brown,[4] Ann M. Flenniken,[5,6] Paul Flicek,[1] Antonella Galli,[7] Hamed Haseli,[1] Martin Hrabě de Angelis,[3,8,9] Jong Kyoung Kim,[10] K. C. Kent Lloyd,[11] Colin McKerlie,[5,6,12] Hugh Morgan,[4] Stephen A. Murray,[13] Lauryl M. J. Nutter,[5,12] Patrick T. Reilly,[14] John R. Seavitt,[15] Je Kyung Seong,[16] Michelle Simon,[4] Hannah Wardle-Jones,[7] Ann-Marie Mallon,[4] Damian Smedley,[2] Helen E Parkinson,[1] and the IMPC consortium.

[1]European Molecular Biology Laboratory, European Bioinformatics Institute (EMBL-EBI), Wellcome Genome Campus, Hinxton, Cambridge CB10 1SD, UK.

[2]Clinical Pharmacology, William Harvey Research Institute, School of Medicine and Dentistry, Queen Mary University of London, Charterhouse Square, London EC1M 6BQ, UK.

[3]German Mouse Clinic, Institute of Experimental Genetics, Helmholtz Zentrum München, German Research Center for Environmental Health, Ingolstädter Landstrasse 1, 85764, Neuherberg, Germany.

[4]Medical Research Council Harwell (Mammalian Genetics Unit and Mary Lyon Centre), Harwell, Oxfordshire OX11 0RD, UK.

[5]The Centre for Phenogenomics, Toronto, Ontario M5T 3H7, Canada.

[6]Mount Sinai Hospital, Toronto, Ontario M5G 1X5, Canada.

[7]Wellcome Trust Sanger Institute, Cambridge CB10 1SA, UK.

[8]German Center for Diabetes Research (DZD), Ingolstädter Landstr. 1, 85764 Neuherberg, Germany.

[9]Chair of Experimental Genetics, School of Life Science Weihenstephan, Technische Universität München, Alte Akademie 8, 85354 Freising, Germany.

[10]Department of New Biology, DGIST, Daegu, 42988, Republic of Korea.

[11]Mouse Biology Program, University of California, Davis, California 95618, USA.

[12]The Hospital for Sick Children, Toronto, Ontario M5G 1X84, Canada.

[13]The Jackson Laboratory, Bar Harbor, Maine 04609, USA.

[14]Phenotyping Institut Clinique de la Souris, 1 Rue Laurent Fries, 67404 Illkirch Cedex, Alsace, France.

[15]Department of Molecular and Human Genetics, Baylor College of Medicine, Houston, Texas 77030, USA.

[16]Laboratory of Developmental Biology and Genomics, College of Veterinary Medicine, Interdisciplinary Program for Bioinformatics and Program for Cancer Biology, Seoul National University, Seoul, Republic of Korea.

Index

1. **Supplementary Methods** Data sets and orthologue inference.

2. **Supplementary Tables**

3. **References not in the main text**

Supplementary Methods

Viability data and mouse-to-human orthologue inferences

We used mouse viability data collected by the International Mouse Phenotypic Consortium (IMPC, www.mousephenotype.org) using a dedicated embryonic pipeline. The current dataset corresponds to Data Release (DR) 7.0., which includes data for 1,751 genes previously published (Dickinson et al. 2016, DR 4.0) as well as data collected since then. Viability data by the IMPC is analysed as defined in IMPRESS (the International Mouse Phenotyping Resource of Standardised Screens, https://www.mousephenotype.org/impress/), based on a minimum of 28 pups screened before weaning and ascertains absence of knockout (null) homozygote pups to classify the gene as essential. Thus, lethal lines are defined as those with an absence of null homozygous pups, or lacking a heartbeat, while subviable lines are those with fewer than 12.5% homozygous pups (half of the 25% expected; $P < 0.05$, binomial distribution). Viable mouse lines are those for which homozygote (null and wild type) and heterozygote pups are observed in normal Mendelian ratios. We filtered out genes for which sample size was insufficient (total pups < 28, $n = 10$ genes), hemizygous genes ($n = 1$ gene), as well as those with conflicting calls (genes that appear in more than one viability category, $n = 29$ genes), resulting in a set of 4,237 genes, of which 1,052 had a lethal phenotype, 383 a subviable phenotype, and 2,802 a viable phenotype (25%, 9% and 66%, respectively). We compared these results with those obtained in studies of human cell lines, in which gene essentiality was determined by observing cell proliferation (cell viability) after a gene was knockout, of a total of approximately 18,000 genes. We used the human cell datasets from Blomen et al. (2015), Hart et al. (2015) and Wang et al. (2015), involving 2, 5 and 4 human cell lines, respectively.

In order to compare mouse and human viability data sets based on IMPC mouse data and human cell studies, first we unified all human gene names in all data sets for the most recent HGNC approved symbol. To do this, we used the Multi-symbol Checker tool available through the HGNC website. All symbols from the literature (Hart et al. 2015, Wang et al. 2015) that mapped to more than one identifier (previous symbol, synonym) were discarded (~4% of the genes). When Ensembl identifiers as well as symbols were provided (Blomen et al. 2015), we obtained HGNC identifiers by accessing the Ensembl REST API using a custom script and the Multi-symbol Checker tool available through the HGNC website; conflicting identifiers were discarded. This resulted in a combined dataset comprising 18,862 genes. We then assigned the labels "essential" and "non-essential" to each cell line value, following the thresholds established in the original studies for each cell line. We then classified a gene as essential if it was essential for cell viability in more than 50% of the cell lines where the gene had been studied, when it had been studied in more than 50% of the cell lines (equivalent to ≥ 6 cell lines; 1,189 genes discarded). Second, we inferred mouse-to-human orthologues using custom scripts and the human-mouse orthologue file compiled by HCOP (Eyre et al. 2007), which we downloaded on 6 March 2018 from the HGNC website (HUGO Gene Nomenclature Committee; Gray et al. 2015). Orthologue predictions compiled by HCOP were obtained using 12 inference methods (eggNOG, Ensembl Compara, HGNC, HomoloGene, Inparanoid, NCBI Gene Orthology, OMA, OrthoDB, OrthoMCL, Panther, PhylomeDB and TreeFam). We kept inferences having a prediction based on 5 or more inference methods, which resulted in a dataset containing 4,087 mouse-to-human orthologues with IMPC viability information. This IMPC mouse-to-human orthologue viability dataset comprised 1,044 lethal, 380 subviable and 2,663 viable genes (26, 9 and 65%, respectively), thus resulting in nearly identical proportions to those obtained from the entire viability dataset. Of these, 4,028 genes had been analysed in the human cell studies. With these data, we obtained genes necessary for organism and cell viability (Table 1).

Obtaining mouse orthologues for genes in non-model species

In order to explore the opportunities for IMPC data to contribute to conservation and evolutionary biology studies of mammal species, we selected studies that explored functional adaptations in non-model species based on genomic data. The selected species included the African cheetah (*Acinonyx jubatus)*, the gorilla *(Gorilla gorilla)*, the grey wolf (*Canis lupus*), the giant and red pandas (*Ailuropoda melanoleuca* and *Ailurus fulgens*), the Iberian lynx (*Lynx*

pardinus), the polar bear (*Ursus maritimus*), and the Tasmanian devil (*Sarcophilus harrisii*), and explored the opportunities for the IMPC to contribute functional knowledge.

We retrieved mouse orthologues for the giant panda, the grey wolf, the western gorilla, the Tasmanian devil, the polar bear, and the Iberian lynx using MetaPhOrs (Pryszcz et al. 2011), applying an Orthology Consistency Score (CS) cut-off of 0.5 (ftp://phylomedb.org/metaphors/release-201601/orthologs, last accessed 06 September 2017) (Supplementary Table S4). MetaPhOrs provided mouse orthologues that we converted to Ensembl protein identifiers using the MetaPhOrs cross-reference file, ext2meta.txt.gz, and then to MGI (Mouse Genome Informatics) identifiers by means of the BiomaRt R package (Durinck et al. 2009). MetaPhOrs is a publicly available repository of phylogeny-based orthology and paralogy predictions computed using seven homology prediction services (COG, eggNOG, Ensembl Compara, Fungal Orthogroups, OrthoMCL, PhylomeDB and TreeFam) and based on 705,123 phylogenetic trees for 829 genomes. We used these data sets to retrieve the number of IMPC phenotypes that could be associated to these species via their mouse orthologues (Figure 2).

We then obtained specific sets of genes of interest from the different species, including infertility genes in the African cheetah (Dobrynin et al. 2015), loss-of-function (LoF) genes and disease genes in gorillas (Xue et al. 2015), positively selected genes in the polar bear (Liu et al. 2014), wolf genes potentially under selection (42K SNP array, Schweizer et al. 2016a), wolf genes with nonsynonymous mutations significantly correlated with environmental variables (resequencing data of 1,040 loci, Schweizer et al. 2006b), and giant and red panda genes involved in convergent evolution to a bamboo-rich diet (Hu et al. 2017). We inferred orthologous genes using Ensembl BioMart (Ensembl release 92) to obtain ENSMUSG (mouse) identifiers. When BioMart provided no orthologue, we used the Ensembl website, which contains additional information. We used the ENSMUSG identifiers to obtain MGI identifiers in the MGI database. Ensembl mouse gene identifiers (ENSMUSGXXXXX) or MGI identifiers (MGI:XXXXX) can both be used to query the IMPC (www.mousephenotype.org) and MGI (http://www.informatics.jax.org/) databases to obtain phenotype data.

Supplementary Tables

Supplementary Table S1 Test for the differences in the distribution of gorilla LoF alleles in the 3 viability categories obtained for IMPC mice (alternative hypothesis, H_1) at the significance level of 0.05 (A). A test in accordance to 2 viability categories was also performed (B). Gbb, mountain gorillas; Gbg, eastern lowland gorillas; Ggg, western lowland gorillas.

A) χ^2 goodness-of-fit test

	**Lethal**	**Subviable**	**Viable**	***P* value**
**IMPC**	1,052	383	2,802	
**Gbb**	5	3	13	0.7313
**Gbg**	4	3	14	0.6588
**Ggg**	5	6	26	0.1303

B) Exact binomial test

	**Lethal**	**Viable**	***P* value**
**IMPC**	1,052	2,802	
**Gbb**	5	13	1.0000
**Gbg**	4	14	0.7941
**Ggg**	5	26	0.0905

Supplementary Table S2 Exact binomial test for the differences in the distribution of gorilla LoF alleles in the 2 viability categories obtained for human cell lines (H_1) at the significance level of 0.05. See Table S1 for gorilla population abbreviations.

	**Essential**	**Viable**	***P* value**
**Human cells**	1,568	16,105	
**Gbb**	4	55	0.8177
**Gbg**	3	58	0.3694
**Ggg**	8	102	0.7362

Supplementary Table S3 Human (a) and mouse orthologues (b) of gorilla genes with homozygous LoF alleles and their association to essentiality based on human cell studies (a) or IMPC and MGI data (b), used in Figure 1.

(A) Human orthologues

Population	Essential	Non-essential	No data
**Gbb**	1	13	1
**Gbg**	0	14	0
**Ggg**	6	68	7
**Gbb;Gbg**	1	14	2
**Gbb;Ggg**	0	4	1
**Gbg;Ggg**	0	6	0
**Gbb;Gbg;Ggg**	2	24	2

(B) Mouse orthologues

	Lethal		Viable		No data
**Population**	**IMPC**	**MGI**	**IMPC**	**MGI**	
**Gbb**	1	0	3	5	7
**Gbg**	0	3	3	1	4
**Ggg**	4	12	19	20	34
**Gbb;Gbg**	3	2	4	7	12
**Gbb;Ggg**	0	1	0	1	4
**Gbg;Ggg**	0	0	1	4	3
**Gbb;Gbg;Ggg**	1	7	6	4	16

Supplementary Table S4 Number of genes identified in a number of selected species and their mouse orthologues as retrieved from MetaPhOrs, and the number of associated phenotypes in knockout mice collected from the IMPC database, DR6.0.

	**Genes identified**	**Genes with mouse orthologues***	**Unique mouse orthologues**	**IMPC phenotyped****	**With IMPC significant phenotype association**
**Iberian lynx**	23,210	17,791	17,206	4,251	3,372
		77%		*25%*	*79%*
**Polar bear**	21,085	14,931	14,903	3,750	2,976
		71%		*25%*	*79%*
**Tasmanian devil**	18,779	15,529	16,069	3,898	3,090
		83%		*24%*	*79%*
**Western gorilla**	20,404	17,105	17,291	4,374	3,466
		84%		*25%*	*79%*
**Grey wolf**	19,762	17,638	17,893	4,460	3,536
		89%		*25%*	*79%*
**Giant panda**	19,306	17,548	18,226	4,474	3,544
		91%		25%	*79%*

* More than one gene may match to the same mouse orthologue

** Includes young adult phenotypes, viability and fertility data

References not in the main text

Durinck S, Spellman PT, Birney E, Huber W (2009) Mapping identifiers for the integration of genomic datasets with the R/Bioconductor package biomaRt. Nature Protocols, 4, 1184.

Eyre TA, Wright MW, Lush MJ, Bruford EA (2007) HCOP: a searchable database of human orthology predictions. Briefings in Bioinformatics, 8, 2-5.

Gray KA, Yates B, Seal RL, Wright MW, Bruford EA (2015) Genenames.org: the HGNC resources in 2015. Nucleic Acids Research, 43, D1079-D1085.

Pryszcz LP, Huerta-Cepas J, Gabaldon T (2011) MetaPhOrs: orthology and paralogy predictions from multiple phylogenetic evidence using a consistency-based confidence score. Nucleic Acids Research, 39, e32.
